# Interictal Epileptiform Discharges and the Quality of Human Intracranial Neurophysiology Data

**DOI:** 10.3389/fnhum.2020.00044

**Published:** 2020-03-03

**Authors:** Simon G. Ammanuel, Jonathan K. Kleen, Matthew K. Leonard, Edward F. Chang

**Affiliations:** ^1^Department of Neurological Surgery, University of California, San Francisco, San Francisco, CA, United States; ^2^Department of Neurology, Weill Institute for Neurosciences, University of California, San Francisco, San Francisco, CA, United States

**Keywords:** epilepsy, interictal discharges, intracranial recordings, data quality, signal processing

## Abstract

Intracranial electroencephalography (IEEG) involves recording from electrodes placed directly onto the cortical surface or deep brain locations. It is performed on patients with medically refractory epilepsy, undergoing pre-surgical seizure localization. IEEG recordings, combined with advancements in computational capacity and analysis tools, have accelerated cognitive neuroscience. This Perspective describes a potential pitfall latent in many of these recordings by virtue of the subject population—namely interictal epileptiform discharges (IEDs), which can cause spurious results due to the contamination of normal neurophysiological signals by pathological waveforms related to epilepsy. We first discuss the nature of IED hazards, and why they deserve the attention of neurophysiology researchers. We then describe four general strategies used when handling IEDs (manual identification, automated identification, manual-automated hybrids, and ignoring by leaving them in the data), and discuss their pros, cons, and contextual factors. Finally, we describe current practices of human neurophysiology researchers worldwide based on a cross-sectional literature review and a voluntary survey. We put these results in the context of the listed strategies and make suggestions on improving awareness and clarity of reporting to enrich both data quality and communication in the field.

## Introduction

Intracranial electroencephalography (IEEG) transcends many physical limits of scalp electroencephalography (EEG) and magnetoencephalography (MEG) by recording signals directly from brain tissue. Rapid advances in computer processing in recent decades has expanded software and hardware capacities, enabling simultaneous recordings from hundreds of intracranial sites at microsecond precision. These increases in temporal and spatial resolution have enhanced diagnostic precision for seizure localization (Andrews et al., 2019; Cuello Oderiz et al., 2019) and led to an acceleration in human intracranial neurophysiology research (Chang, 2015; Parvizi and Kastner, 2018).

**Figure 1:**
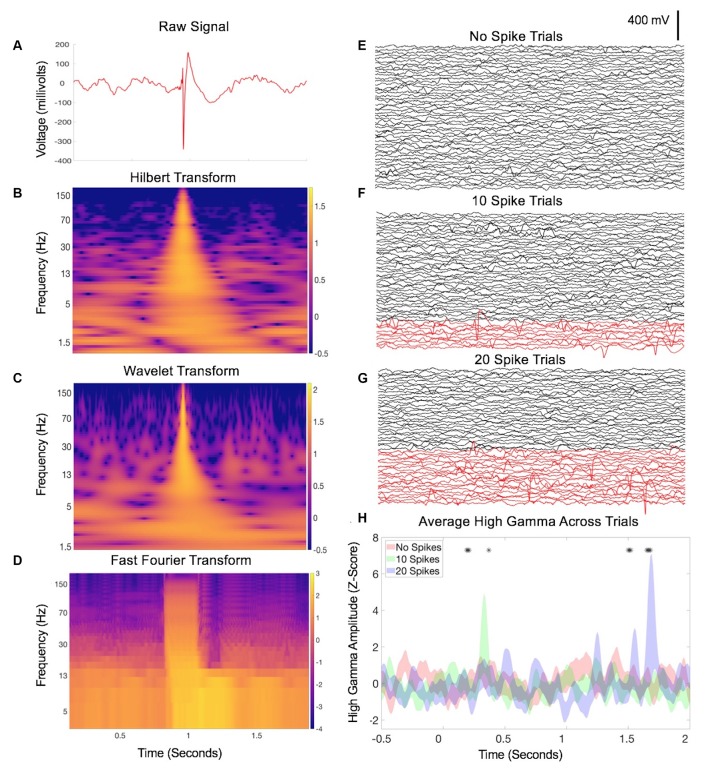
Interictal epileptiform discharges (IED)-related data contamination. **(A)** Example of an IED from a single channel during a 2-s intracranial electroencephalography (IEEG) recording. Classic features are apparent including a sharp, large-amplitude displacement of voltage and an after going slow-wave, otherwise with a relatively normal baseline mix of frequencies before and afterward. **(B)** Hilbert transform spectrogram of the data in **(A)**. Note the transient but substantial increase in power across nearly all frequencies, due to the sharp component of the waveform, and a subtle sustained increase in low-frequency power related to the after going slow-wave. **(C)** Wavelet transform spectrogram of the data in **(A)**, with similar findings as in **(B)**. **(D)** Fourier transform of A (Mitra and Bokil, 2007; Chronux Home, 2019) with 0.25-s overlapping windows, sliding point-by-point to provide similar time resolution as **(B,C)**. Similar findings as in **(B,C)**, with an additional duration of the power increase in the faster frequencies due to the nature of the consistent time window across frequencies for the FFT calculation. Panels **(E–G)** each display IEEG data from 50 trials, recorded from a single channel during a speech listening task (one pre-recorded sentence played aloud for each trial starting at time zero). In panel **(F)**, 10 trials were swapped with trials that contained IEDs, shown in red. A hybrid of manual and automated approaches (Baud et al., 2018) was used. Panel **(G)** increases this to 20 trials with IEDs. Panel **(H)** shows the average high gamma across trials in each group (Hilbert transform, 50–200 Hz) from one electrode contacting the inferior temporal gyrus that was not truly modulated by the sentence-listening task. Asterisks denote time points during which one of the latter groups deviates significantly from baseline (two-way repeated-measures ANOVA, *p* < 0.05). As the proportion of trials with IEDs increases, additional falsely positive timepoints emerge.

Along with emerging computational tools and capacities for massive dataset analysis, the wealth of neuroscientific opportunities and potential discoveries is promising. However, signal analysis on human intracranial recordings invokes inherent pitfalls that are presumably addressed but minimally acknowledged in many neurophysiological studies of human patients—namely, interictal epileptiform discharges (IEDs). IEDs are transient bursts of activity produced by groups of neurons that are pathologically connected due to epilepsy, resulting in distinct and prominent waveforms during IEEG recordings (Figure 1A). This Perspective will draw attention to IED hazards, potential effects on common analysis strategies, and describe common strategies to avoid them so that the growing wave of discoveries in human neurophysiology continues to advance hopefully without missteps.

## The Nature of IED Hazards in Human IEEG Neurophysiology

The crux of our view is that the paradigm of human intracranial neurophysiology exposes well-meaning scientists to risks of erroneous results due to two main factors:

(1) Electrodes are implanted in regions of the human brain that are deemed likely to reveal neurophysiological signatures of epilepsy in both ictal and interictal contexts—which are often sharp large-amplitude waveforms.
(2) Signal processing analyses that are commonly used for human neurophysiology are exquisitely sensitive to the sharp large-amplitude waveforms described in #1, which cause spurious results.

Therefore, IEEG datasets commonly contain electrical signatures of epilepsy that convey risk for distorted results when included in common signal analyses such as power and coherence measures, and related methods such as power-phase co-modulation (Kramer et al., 2008). One way to account for this problem is that many neural signals processing methods such as Fourier, Wavelet, and Hilbert-based analyses assume a sinusoidal data substrate (van Drongelen, 2018). Convolving large amplitude waves or sharp deflections (large or even small amplitude) therefore predisposes to representations of many frequencies that can be largely spurious, since many consecutive sinusoidal functions may fit these elements of the waveform. In Figures 1B–D, we show examples of how IEDs can easily misrepresent neurophysiological signals in this manner across commonly used spectral methods of Hilbert, Wavelet, and Fourier transforms. In lower frequency bands, both the sharp and slow-wave components of IEDs can induce a power increase in any frequency that fits these features. In higher frequency bands, this striking power burst across vast stretches of continuous frequency levels becomes even more obvious and can be referred to as a ringing or spectral leakage (Scheffer-Teixeira et al., 2013). This can be evident across the entire high gamma band (50–200 Hz or other similar range), a concerning issue given many neurophysiology laboratories utilize high gamma activity given its potential value as a surrogate for local neural activity (Ray et al., 2008). The example in Figure 1H demonstrates how cumulative inclusion (Figures 1E–G) of trials with spikes adds spurious variability (risking a false negative) or otherwise influences statistical significance (risking a false positive).

This problem could be more pervasive than in EEG or MEG recordings due to direct neural tissue contact, which can convey larger spike amplitudes and sharper deflections particularly in the case of IEDs, contaminating neurophysiological analyses. Furthermore, intracranial electrodes are specifically placed in regions that are likely to be clinically associated with the epileptic seizure focus, leading to strong and/or frequent IEDs in some IEEG datasets.

Anecdotally, most researchers in human neurophysiology would agree that data contamination by IEDs is common knowledge, although the impact of this may vary for certain types of analyses (Meisler et al., 2019). In fact, one would anticipate that many research labs have strategies in place to circumvent, or at least minimize this problem. However, when practically assessed, this problem and its potential ramifications may be much more pervasive than anticipated. A third complicating factor may illustrate how this can be so:

(3) Neurophysiology researchers (especially early trainees such as students and postdoctoral researchers) may not receive direct training in the identification of IEDs or electrical artifacts. In addition, the spectrum of potential IED morphologies, and how their distinct features can be expected to contaminate signal analyses, may not be engrained within standard training.

As a further complication, the inter-rater agreement for IED detection is surprisingly poor, even among fully trained epileptologists (Barkmeier et al., 2012; Janca et al., 2015).

## Strategies for Handling IEDs

There are a variety of approaches when encountering IEDs in data, which may or may not depend on their rate and spatial extent (for example, a researcher may not be inclined to curate and “clean” a dataset for one spike per minute but may for 10 spikes per minute). We distilled the potential approaches of researchers into four main strategies. The first is to manually identify and remove any trials or periods during which IEDs occur, referred to here as Strategy 1. The data are screened through by examining plots of the recorded data with or without some pre-processing (notch and/or bandpass filtering) and segments of data that contain IEDs are marked so that any trials that overlap with these segments can be left out of the analyses or converted to missing values.

Certain researchers have received formal clinical training in reading EEG/iEEG, though this is less common for many non-clinical researchers who are trained through graduate-science academic tracks. While many have received either didactic and/or one-on-one training on how to identify and remove IED trials from datasets, some may have not. Complicating the matter, IEEG datasets are far from standardized because of recording differences: the layout of seizure-generating networks differs for each patient resulting in variable numbers of electrodes, not to mention individual differences in neuroanatomy and the laterality of the implantation. There are mixed contact modes (grids, strips, depths) of varying densities, along with customized ordering of the channels (montages). These factors all lead to increased difficulty in the interpretation of IEEG analyses, particularly for those who have not received adequate training (formal or informal) for IED identification in these recording layouts. Lastly, as the duration of recordings increases, Strategy 1 diminishes in practicality due to time and effort constraints.

Computerized spike detection algorithms have been developed in recent decades to equip the next generation of scientists with effective and standardized spike-detecting abilities, save time, and circumvent human error. The use of IED detectors and removal of affected trials/data in an automated fashion constitutes another approach, which we will call Strategy 2. Fortunately, IEDs tend to have the features that are often salient to many algorithms: large-amplitude, sharp components, and at times with pathophysiological high-frequency oscillations that are less-often encountered in the normal brain (though see Frauscher et al., 2018b). Ideally, an algorithm will maximize both sensitivity and specificity, while decreasing or removing the contribution (supervision, such as threshold-setting) required from the user. It is difficult to satisfy this wish-list completely, however, and thus many algorithms have been developed using a variety of automated and unsupervised approaches. These include EEG derivations (White et al., 2006), line-length and power transforms (Esteller et al., 2001; Bergstrom et al., 2013), adaptive directed transfer functions (Wilke et al., 2009), spatial filters (Liu et al., 2015), and spike-template matching algorithms such as spatiotemporal regression (Tousseyn et al., 2014) and non-negative matrix factorization (Baud et al., 2018) among many other approaches. All tools have sensitivity and specificity trade-offs; no approach provides 100% certainty, and this is further complicated by the lack of a dependable human gold standard. Specifically, the poor inter-rater agreement of manual detection among highly trained individuals noted above, and the “quantitative gray-zone” of small questionable IEDs they may ignore, are fundamental caveats for algorithm testing. Lastly, while nearly all new methods are compared to manual detection or one other automated method, a broad comparison across most or all automated methods is difficult due to technical challenges of implementing each in turn on a sufficiently large dataset (Westover et al., 2017). Nevertheless, the automated removal of interictal spikes using unsupervised and/or supervised approaches can save time, approach standardization, and improve neurophysiology data quality.

As a result of the caveats (and potential fallacies) of automated detection algorithms, some researchers who employ them may hesitate to allow full discretion to this mechanism. Since automated detection approaches can drastically increase efficiency for the bulk of obvious detections, a third strategy (Strategy 3) is a hybrid approach of Strategies 1 and 2, in which the automated detections are also manually screened (often in this order, although the opposite order or multiple iterations can also be applied). An example of this hybrid is through the use of distributions of morphological features (e.g., slope, power measures, etc.) to which a threshold can be applied, followed by manual inspection for potential false positives and/or negatives.

Given the complexity of gathering this precious data and its associated scarcity, a drawback common to Strategies 1, 2 or 3 is that trial removal reduces statistical power. Accordingly, another bias—toward keeping more trials—allows more potential spurious signal results to be introduced, adding the risk of false negatives and positives as described above.

The preference to keep as many trials as possible introduces a different strategy for dealing with IEDs: agnosticism, in which IEDs are ignored (not assessed) and no trials are removed (Strategy 4). One might naturally assume that data afflicted by IEDs will be infrequent and random enough to where affected timepoints will blend into the background of an averaged signal, adding variability but not significantly skewing the results. Meanwhile, task-related neurophysiological signatures would hopefully emerge from the analysis and prevail, if present, by virtue of their consistency across trials. As a signal-to-noise problem, this assumption may be valid for infrequent IEDs, especially with a strong experimental effect size (though an additional safety layer of using nonparametric statistics may be advised). However, more frequent (Figures 1E–H) and/or larger or sharper IEDs can undermine this approach. Nevertheless, robust statistical power is always preferred, and often requires large numbers of trials that are more likely attained in the agnostic approach of Strategy 4, particularly for subtle effect size. In fact, one recent study (Meisler et al., 2019) formally assessed whether manual, automated or no removal of IEDs affected their neurophysiological findings in an episodic memory task—they found no clear effect of any approach, though emphasized the importance of sufficient numbers of trials. On a related note, newer machine learning analyses require large volumes of training data to build accurate models—these along with deep learning approaches can learn to differentiate between normal neurophysiologic signals and pathological IED waveforms, provided the previous training data is accurately labeled (often manually). Thus, the agnostic strategy has a certain appeal (including low-effort) and could be a good default for some studies, assuming there is enough data to employ it.

## Current Practices Among IEEG Researchers

**Figure 2:**
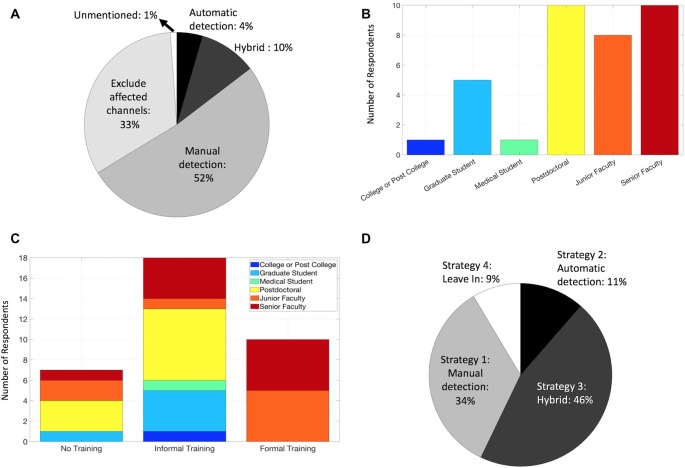
Current practices for handling IEDs among IEEG researchers. **(A)** Methods of handling IEDs as reported by 2018 manuscripts that matched our PubMed search and screening criteria. **(B)** Career levels among survey respondents. **(C)** Training regarding identification and/or removal of IEDs among survey respondents. **(D)** Strategy used for handling IEDs among survey respondents.

With these diverse general strategies in mind, how are IEDs currently handled by human neurophysiology researchers? We assessed this question of “Current Practices” by first summarizing how researchers describe their methods in published literature. We searched PubMed using two broad queries: (*intracranial AND eeg*; *electrocorticography*) and filtered the results to include only studies published in 2018. We limited the search results by examining each article (613 unique articles) and included only those that measured intracranial neurophysiological signals in humans and appeared to make conclusions regarding normal neurophysiology (91 total). Although these search conditions are not exhaustive, they provide a contemporary snapshot of diverse groups worldwide studying normal human neurophysiology *in vivo*. We found that the majority of these publications alluded to using manual methods (Figure 2A), including direct identification of IEDs, or a similar (yet more conservative) approach of identifying and excluding electrodes that were covering the seizure foci. Less than 5% of manuscripts used fully automated methods, and none of the included articles used the agnostic approach (Strategy 4). Again, our sample of included articles was limited and these results may not fully represent the field.

Given IED handling methods did not appear to be described in detail in many of the articles, we further assessed our “Current Practices” question by designing an anonymous survey. This survey (approximately ~2 min) consisted of questions regarding career level, whether they were familiar with IEDs in IEEG, and had formal or informal or no training on IED identification and removal, along with a selection of which of the Strategies (1–4) above they tended to use (or other). We emailed the listed corresponding authors of the articles described above (79 total, as some articles overlapped or had multiple corresponding authors) requesting their voluntary participation, which would be anonymized (exempt from IRB requirements per UCSF IRB Office). We asked these individuals to forward the survey to other colleagues and co-workers as well.

We received a 44% response rate, with survey respondents weighted toward post-doctoral and faculty members, likely due to a sampling bias *via* emailing a population of corresponding authors (Figure 2B). Of note, responses from these senior lab members may likely represent the practice of their lab as a whole on their IED Strategy, though this is less applicable for the IED familiarity and training questions. All respondents indicated they were familiar with IEDs, a reassuring result, though possibly influenced by the nature of the survey. Regarding training on recognizing IEDs and handling method(s), the majority were informally trained (Figure 2C), and some were not trained at all. Not surprisingly, those who identified as formally trained were junior and senior faculty, consistent with the fellowship-level clinical requirements for formal EEG training in most contexts. The majority of respondents utilized either a manual approach, whether in isolation or as a manual-automated hybrid (Strategies 1 and 3; Figure 2D), generally comparable to the literature review (Figure 2A) as expected. Interestingly, we found no articles that explicitly indicated the use of the Strategy 4 (“agnostic”) despite 9% of respondents identifying as such, and the incorporation of automated methods (Strategies 2 and 3) had a larger representation in the survey responses (55%) than the literature review (14%). These discrepancies could be explained by reporting bias however, given the different contexts of manuscript methods vs. a direct survey, and sampling bias (survey response rate).

## Further Considerations

While this article focuses primarily on IEDs, the hazards and strategies described herein can and should be extended to other electrical or non-physiologic artifacts (cable movements, electrode pops, amplifier saturation, etc.), since they can involve similar sharp large-amplitude deflections. Regarding channel exclusion, channels with abundant IEDs are particularly problematic for automated methods that rely on background estimates. Furthermore, if normal neurophysiology is to be studied, these channels and any that are known to be in lesional tissue should be excluded outright (Frauscher et al., 2018a); this practice was reflected in about one-third of manuscripts in our literature review (Figure 2A).

Regarding proper experimental control, it is often paramount that IED-marking is performed while blinded to the task events and experimental conditions (most relevant for manual Strategies 1 and 3), to prevent biases that could influence study results (e.g., removing trials with IEDs more often from one condition than another). Regarding potential confounds in trial-based studies, IEDs are often assumed to occur unpredictably, even to the point of randomness: this assumption is favorable if leaving IEDs in the data (Strategy 4) since false-positives and -negatives would be diminished through trial averaging which improves the neurophysiological signal-to-noise ratio. However, it should be noted that the timing of IEDs may not necessarily be random in a behavioral task. Task-dependent modulation of the timing or amount of IEDs has been described (Matsumoto et al., 2013), which could potentially confound results by preferentially weighting spurious results in certain trial segments more than others. This would argue against the use of Strategy 4, though again, the conundrum of trial numbers and statistical power can be problematic as noted above.

Lastly, apart from signal processing implications, it is worth mentioning that IEDs can also transiently disrupt the local neural dysfunction of the region in which they occur (Krauss et al., 1997; Kleen et al., 2013; Horak et al., 2017; Ung et al., 2017). This could lead to cognitive errors that could influence trial-based and other analyses, posing a separate argument for the exclusion of trials with IEDs when making conclusions regarding “normal” cognitive processing.

## Conclusions

The acceleration of human intracranial neurophysiology conveys great excitement for impending discoveries and capabilities, including expanding the basic neurosciences, improving clinical therapeutics and the development of brain-machine interfaces. However, IEDs pose pitfalls of spurious results, difficult to avoid by the nature of the *in vivo* epileptic tissue from which data is recorded. Increased vigilance is needed to avoid IEDs in data if/when appropriate, which can be afforded by the consideration and use of the strategies listed above. We also suggest that mid-level and senior researchers should make attempts to enhance and provide standardized training presentations or simulations in their labs for IED identification, detection, and removal methods. This will equip younger researchers with an important skill set to understand and constructively scrutinize their own data and that of others. Furthermore, improvements in scientific communication are needed (Suthana et al., 2018), such that manuscripts on normal human neurophysiology should clearly convey the approach used for handling IEDs and its justification in the context of their study. Such practices of increased vigilance and clear communication will hopefully improve reproducibility so that the field can continue its acceleration without foreseeable setbacks.

## Data Availability Statement

The survey data in this article can be downloaded from doi: 10.5281/zenodo.3626105.

## Ethics Statement

The studies involving human participants were reviewed and approved by University of California San Francisco Institutional Review Board. Written informed consent for participation was not required for this study in accordance with the national legislation and the institutional requirements.

## Author Contributions

SA and JK developed the concept of the manuscript, created and distributed the survey, performed the literature search and wrote the manuscript. ML and EC provided significant feedback and edits to the manuscript. All authors read and approved the submitted manuscript.

## Conflict of Interest

The authors declare that the research was conducted in the absence of any commercial or financial relationships that could be construed as a potential conflict of interest.
